# Impact of Early Nutrition on Body Composition in Children Aged 9.5 Years Born with Extremely Low Birth Weight

**DOI:** 10.3390/nu9020124

**Published:** 2017-02-10

**Authors:** Sonja Stutte, Bettina Gohlke, Annika Peiler, Felix Schreiner, Mark Born, Peter Bartmann, Joachim Woelfle

**Affiliations:** 1 Paediatric Endocrinology Division, Children’s Hospital, University of Bonn, 53113 Bonn, Germany; sonja.stutte@ukb.uni-bonn.de (S.S.); bettina.gohlke@ukb.uni-bonn.de (B.G.); felix.schreiner@ukb.uni-bonn.de (F.S.); 2 Department for Orthopedic surgery, Franziskus Hospital, 53545 Linz, Germany; info@krankenhaus-linz.de; 3 Department of Radiology, Children’s Hospital, University of Bonn, 53113 Bonn, Germany; mark.born@ukb.uni-bonn.de; 4 Department of Neonatology, Children’s Hospital, University of Bonn, 53113 Bonn, Germany; peter.bartmann@ukb.uni-bonn.de

**Keywords:** preterm, early nutrition, body composition

## Abstract

To evaluate body composition, metabolism and growth as well as their interaction with early nutrition in former extremely low birth weight infants (ELBW), we assessed qualitative and quantitative nutritional intake during initial hospitalization and infantile growth parameters in 61 former ELBW infants with a birth weight <1000 g. In two follow-up exams, physical and biochemical development were measured at 5.7 and at 9.5 years. At the second follow-up, in addition to biochemical reassessment, body composition was analyzed by dual-energy x-ray absorptiometry (DEXA). Protein intake between birth and discharge was associated with weight gain in the first six months of life (*r* = 0.51; *p* < 0.01). Weight catch-up preceded height catch-up. Protein intake in early infancy correlated highly significantly with abdominal fat mass (*r* = 0.49; *p* < 0.05), but not with lean body mass at 9.5 years (*r* = 0.30; not significant (n.s.). In contrast to nutrient intake, birth weight was associated with lean body mass (*r* = 0.433; *p* < 0.001). Early protein and carbohydrate intake were associated with high-density lipoprotein (HDL)-cholesterol, and early catch-up growth correlated with fasting insulin at follow-up. Stepwise linear regression demonstrated that protein intake predicted fat mass (*p* < 0.05), whereas only gender and birth weight standard deviation score (SDS) contributed significantly to lean body mass variation (*p* < 0.05). Our results suggest an important impact of early nutrient intake on body composition and metabolism in later childhood in ELBW children.

## 1. Introduction

Decreased birth weight can have a persisting impact on metabolic health in later life [1]. In addition to metabolic dysfunction, subjects with low birth weight exhibit an increased risk of cardiovascular impairments in adulthood [2]. Catch-up growth in children with low birth weight has been associated with impaired metabolic parameters in later life [3]. On the other hand, catch-up of head circumference is important for neurological development [4].

Preliminary animal data suggest that the macronutrient content of late fetal or early postnatal nutrition might influence later body composition [5]. Therefore, early life macronutrient composition and total caloric intake currently receive much attention in humans, with special regard to the feeding protocols of infants with low birth weight or born prematurely. Despite the continual improvement of dietary composition and a faster increase in quantitative food intake during initial in-patient stay, premature infants build up an energy deficit which leads to growth failure until discharge from the hospital [6,7].

In part, this might be due to the fact that energy supply in extremely low birth weight infants (ELBWs) often does not reach recommendations from guidelines in the first weeks after birth [8]. Missing these aims has been ascribed to the extent of prematurity. It depends on the clinical condition of the infants between birth and discharge, in particular on the number and severity of infections, the duration of the mechanical ventilation, and on medical treatment.

On the other hand, rapid catch-up growth following an initial growth restraint after preterm birth bears a higher risk of metabolic alterations later in life [9]. This is characterized by a higher abdominal fat mass, arterial hypertension, lower insulin sensitivity, and hyperlipidemia. The association of preterm birth and the occurrence of metabolic syndrome were mainly confirmed in studies in young adulthood [3]. Studies conducted in preterms at a younger age are sparse [10].

We hypothesized that in the special cohort of extremely low birth weight infants, the first signs of metabolic syndrome and altered body composition may be already present in early childhood and that these are related to early life events including the nutritional regimen. 

To establish clear cause/effect relationships between early nutrition and subsequent morbidity, controlled human intervention trials in these groups at risk would be highly desirable. However, even today, feeding of critically ill preterm infants in the neonatal intensive care units (NICUs) remains a balancing act and makes controlled trials with strict adherence to nutritive protocols very difficult if not impossible. We thus performed a longitudinal single-center follow-up study in ELBW infants and focused on the development of height, weight, and metabolism. In addition, we assessed body composition at the last follow-up exam at a mean age of 9.5 years.

## 2. Materials and Methods

### 2.1. Patients

The initial study population consisted of 175 extremely low birth weight infants who were born at the Department of Neonatology at the University Hospital of Bonn in the years 1999–2002, per definition born with a birth weight below 1000 g. A total of 49 children died in the postnatal period, 27 had moved and were not accessible, and 38 refused to take part in the study.

First follow-up examination: A total of 61 children with a mean age of 5.7 ± 0.9 years participated in the first follow-up examination at the Children’s Hospital in Bonn in 2006. The focus of the first follow-up examination was on auxological development. Subjects with chronic or syndromal disorders or with handicaps after severe intraventricular hemorrhages or necrotizing enterocolitis were excluded. For more details see [11].

Second follow-up examination: At the second follow-up in 2010, a total of 39 (22 female, 17 male) healthy children (mean age: 9.5 ± 1.0 years; range: 7.9–11.9) participated in the re-examination. Twenty-two families from the initial cohort could not be convinced to take part in the follow-up. The main reasons for not participating were having moved to more distant locations and unwillingness to travel, fear of stress to the children or refusal to participate in the dual energy X-ray absorptiometry (DEXA) analysis. 

All but eight of the children (six male) were prepubertal. The children’s parents and the children themselves were asked for written informed consent and agreed to the study. The protocol was approved by the Medical Ethics Committee of the Faculty of Medicine at the University of Bonn and the Federal Agency for Radiation Safety (Ethics approval code EK 115/06).

### 2.2. Measurement of Nutritional Intake

Nutritional intake from birth until discharge was collected retrospectively from patients’ files. The following components were analyzed: total daily energy-, carbohydrate-, lipid- and protein-intake. For analysis, we calculated the average daily nutritional intake of each component over the whole in-patient stay, expressed in g/kg/day. Due to the fact that our cohort consisted of ELBW infants, strict adherence to a detailed nutritional protocol was not possible, in particular in critically ill infants. Several ELBW infants had to switch from oral to parenteral nutrition during clinical deterioration; therefore, the amino acid composition of the protein intake differed between patients and was not part of this analysis. Not prescribed, but actually taken amounts of nutrients were used for calculations.

### 2.3. Measurement of Auxological Parameters

Growth parameters (weight and height) at birth and during postnatal in-patient stay were gathered retrospectively from patients’ files. Data concerning growth during infancy were collected from regular paediatric screening examinations. Children were seen at a mean age of one, two and four years as part of the recommended German prevention program. The first follow-up examination as part of the study protocol, was performed at a mean age of 5.7 years. Mean age at the second follow-up examination was 9.5 years. All children underwent standardized auxological measurements which were always conducted by the same person. All children were weighed with the same scales while wearing underclothes. Height was determined by the same fixed stadiometer in all cases. For analysis, age was corrected for preterm birth until two years of age. Weight and height standard deviation-scores were calculated using German reference data [12,13]. Body-mass-index in kg/m² (BMI) was standardized in agreement with national reference data [14]. To illustrate weight and height gain over the whole period, we calculated the change in weight/BMI, resp. height standard deviation score (SDS) between successive examinations.

In addition to the outlined auxological parameters, we measured triceps’ skinfold thickness with a calibrated calliper according to standardized reference manuals. For analysis, reference data for standard deviation scores were derived from Gerver and coworkers 2001 [15].

### 2.4. Measurement of Body Composition

Measurement of body composition only took place at the second follow-up examination with a mean age of 9.5 years. Lean body mass, total and regional (abdominal, hip) fat mass were measured using dual-energy x-ray absorptiometry (Lunar DXA, GE Healthcare). The DXA scans were all performed with the same standard dose apiece (76 kV/0.15 mA/0.4 µGym²). During the examination, every child wore shorts and lay in a standardized position on the back. We did not have to use sedatives. For analysis, reference data for standard deviation scores were provided from GE Lunar Body Composition Software (enCORE 2010; Version 13.31, GE healthcare, Madison, WI, USA).

### 2.5. Biochemical Analyses

Venous blood samples for laboratory analysis were collected from all children between 08:00 a.m. and 10:00 a.m. in a fasting state. Levels of insulin were measured with an Immulite 2000 analyzer (Pharmacia and Upjohn Diagnostics AB, Uppsala, Sweden) with the lowest detection limit at 2.0 µU/mL). Total cholesterol, HDL- and low-density lipoprotein (LDL)-cholesterol, triglycerides were measured with Vista 3000T (Siemens Healthcare, Newark, NJ, USA).

### 2.6. Definitions

We defined “catch-up growth” as an increase of weight or length of >1 SDS during an interval of six months.

### 2.7. Statistical Analysis

Statistical analysis was performed using the SPSS software (SPSS, Version 20, IBM Deutschland GmbH, 71137 Ehningen, Germany). Non-parametric tests were used whenever data were not normally distributed. Relationships between variables were examined by non-parametric Spearman’s rank correlation, partial correlations or linear regression analysis. Linear regression analyses were calculated with lean body mass, fat mass, HDL-cholesterol and fasting insulin as dependent variables and sex, birth weight, early macronutrient intake, weight development and pubertal stage as independent variables. Sex was used as a categorical variable in these models. All variables not normally distributed were log-transformed in these models. Significance was defined as *p* value < 0.05; a statistical trend was assumed for *p* values between 0.05 and 0.1.

## 3. Results

### 3.1. Growth from Birth until 9.5 Years of Age

A comparison of weight and height development of our study cohort is shown in Table 1. From birth onwards, weight-SDS declined continuously to an averaged minimum of −2.2 SDS at discharge. The highest gain in weight took place during the first six months after discharge. Mean weight-SDS at 0.5 years of age almost reached the initial value at birth. Between 0.5 and 5.7 years, weight-SDS stabilized at around −1 SDS followed by a further improvement in weight gain until the second follow-up-examination.

The time pattern of height development differed from weight development: Height-SDS also declined from birth onwards. In contrast to weight development, the lowest mean height occurred at 0.5 years of age. The highest height catch-up took place between 0.5 and 1 year of age (+4.28 SDS) followed by a slower catch-up growth in height up to 2 years of age. At 9.5 years of age, target height-SDS (mean: 0.1 SDS; range: −2.25 to +1.58) was reached by one third of the cohort.

BMI-SDS declined until 1 year of age and continually increased up to the time of the second follow-up examination.

### 3.2. Relationship between Early Postnatal Nutrition and Auxological Parameters

The higher the average protein intake between birth and discharge from hospital, the more pronounced was the subsequent weight gain. This correlation was particularly present in the time interval of catch-up growth from discharge to 0.5 years of age (*r* = 0.505; *p* < 0.01). Weight development during further follow-up is depicted in Table 1, exhibiting only minor changes in weight SDS between 0.5 and 5.7 years, followed by a moderate increase in weight between 5.7 and 9.5 years of age. Correlation coefficients between early protein intake and delta weight SDS between subsequent follow-up examinations were, between 0.5 and 1.0 year −0.146 (n.s.); between 1.0 and 2.0 years −0.198 (n.s.); between 2.0 and 4.0 years 0.005 (n.s.); between 4.0 and 5.7 years 0.045 (n.s.); and between 5.7 and 9.5 years 0.440 (*p* < 0.05). None of the other nutritional components (average energy, carbohydrate, lipid intake) correlated with catch-up growth in weight between discharge and 0.5 years of age. Catch-up growth in height between 0.5 and 1 year of age was not influenced by nutritional intake.

### 3.3. Association between Auxological Parameters and Body Composition at 9.5 Years of Age

**Figure 1:**
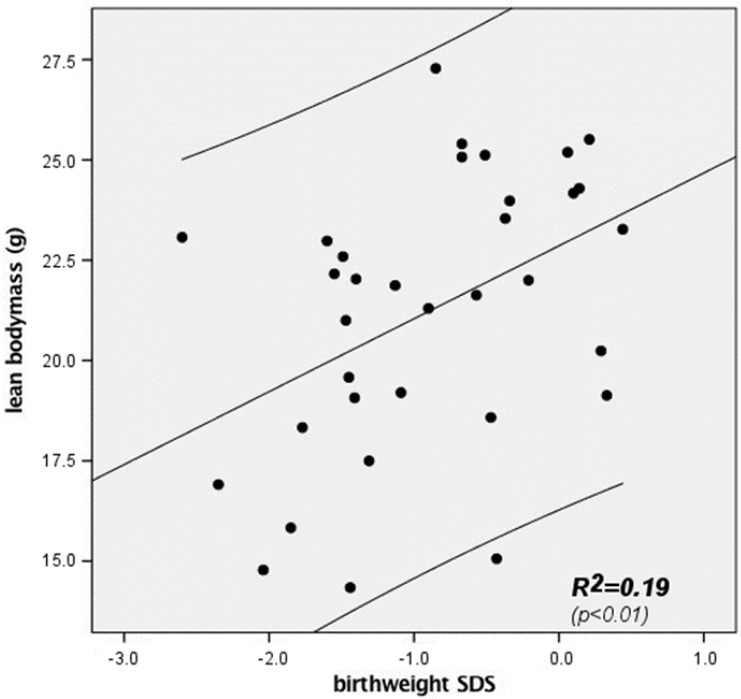
Correlation between birth weight and lean body mass in former ELBW subjects at 9.5 years of age. Lines represent the regression line and the upper and lower limit of the 95% confidence interval (*n* = 35).

As mentioned above, body composition was measured at the second follow-up of our cohort. There was a positive correlation between birth weight-SDS and lean body mass at 9.5 years of age (Figure 1 and Table 2). We found no correlation between birth weight-SDS and fat mass at the same age.

As outlined in Table 1, we found no major changes in weight development between 0.5 and 5.7 years and a moderate weight gain between 5.7 and 9.5 years. Correspondingly, the positive correlation between early protein intake and weight gain between discharge and 0.5 years of age (reported in 3.2) remained significant for comparable calculations with subsequent timepoints. Correlation coefficients between early protein intake and delta weight SDS between subsequent follow-up examinations were: between discharge and 0.5 years 0.505 (*p* < 0.01); between discharge and 1.0 year 0.464 (*p* < 0.05); between discharge and 2.0 years 0.364 (*p* = 0.052); between discharge and 4.0 years 0.211 (n.s.); between discharge and 5.7 years 0.428 (*p* < 0.05); and between discharge and 9.5 years 0.542 (*p* < 0.01).

Absolute height-SDS was positively associated with lean body mass at every measurement from birth onwards. Contrary to weight development, absolute height-SDS did not correlate with fat mass at any point of time. Stepwise linear regression demonstrated that birth weight-SDS and gender contributed significantly to lean body mass variation (*p* < 0.05).

Height development from birth to six months of age correlated significantly with variation in lean body mass (*r* = 0.396; *p* = 0.020), whereas weight from birth to 6 months of age correlated only with variation in fat mass (*r* = 0.411; *p* = 0.022), but not lean body mass (*r* = 0.004; *p* = 0.984). However, in the stepwise multiple regression analysis, neither catch-up in weight nor height contributed significantly to the variation in lean body mass or fat mass, respectively.

### 3.4. Influence of Early Postnatal Nutrition on Body Composition at 9.5 Years of Age

**Figure 2:**
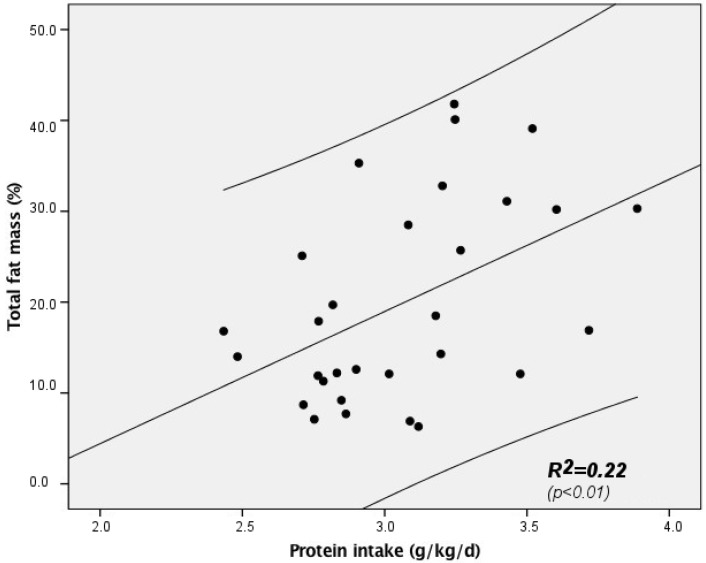
Correlation between early protein intake and fat mass in former ELBW subjects at 9.5 years of age. Lines represent the regression line and the upper and lower limit of the 95% confidence interval (*n* = 30).

Average protein intake during early life correlated significantly with absolute fat as well as abdominal and hip fat mass (see Figure 2 and Table 3).

In addition, triceps’ skinfold thickness also correlated with average protein intake. Lean body mass did not correlate with the amount of protein intake. There was also a positive statistical trend between the average energy intake and absolute, abdominal and hip fat mass as well as to triceps’ skinfold thickness at 9.5 years of age (Table 3). 

Since the stepwise linear regression analysis revealed an interrelation between sex and lean body mass, we re-calculated the correlation coefficients presented in Table 3 and Table 4 as partial correlations with sex as a control variable. Except for the correlation between triceps’ skinfold SDS and protein intake which showed a borderline significant correlation when calculating Spearman’s rank correlation coefficient and which no longer reaches statistical significance with sex as a control variable, correction for sex did not lead to relevant changes (see Supplementary Materials Tables S1 and S2).

### 3.5. Nutritional Intake and Metabolic Parameters at 9.5 Years of Age

Protein and carbohydrate intake during the interval between birth and discharge correlated negatively with HDL-cholesterol at the age of 9.5 years (Table 4). Lipid intake was not associated with any metabolic parameter. Fasting insulin levels and Homeostasis Model Assessment (HOMA) at the age of 9.5 years were highly significantly associated with protein intake, but with none of the other nutritional parameters (Table 4 and supplementary table S2).

Stepwise linear regression demonstrated that protein and carbohydrate intake predicted HDL concentration at 9.5 years (*p* = 0.001), whereas regarding fasting insulin levels, only catch-up in weight between birth and six months of age contributed significantly (*p* < 0.05). 

## 4. Discussion

To the best of our knowledge, this is the first study which demonstrates that quantity and quality of macronutrient intake during the first weeks of life seem to exert a long-term effect on body composition and metabolic health in former very premature ELBW children and that this is already measurable in childhood.

To achieve a sufficient weight and height catch-up, current guidelines focus on an amino-acid enriched formula nutrition [16]. This has been suggested in particular because the intake of protein-enriched formula has resulted in improved cognitive function [17,18,19]. In our study, we demonstrate that early nutrition with increased protein intake might not only be advantageous, but can be associated with an increased risk of metabolic disturbances. We found that early protein intake was associated with an increase in fat mass (absolute, abdominal and hip fat mass) which was already detectable at 9.5 years of age, whereas lean body mass at this age was not affected by protein intake (Table 3, Figure 2). Only birth weight SDS contributed to the observed variability in lean body mass, which is in accordance to previous reports [20,21].

In addition, protein intake explained a significant part of the observed variation of HDL-cholesterol with a decrease in HDL concentration correlating with increasing protein intake. Early protein and energy intake were significantly correlated to weight gain during the first six months of life. In the regression analysis, early weight gain was associated with an increase in fasting insulin level in later childhood. This is in congruence with already reported findings by others [3], although the studied patients in the cohort of Kerkhof et al. were all born at term with the majority being born small for gestational age (SGA) with a birth length < - 2SD.

The exact timing of catch-up growth has been a topic of discussion in the recent literature. In our cohort, weight-SDS decreased dramatically between birth and discharge. This phenomenon has often been described in previous clinical studies [6,7], particularly in extremely low birth weight infants [22]. Although most subjects were appropriate for gestational age at birth, the majority of subjects would have been considered small for gestational age at the expected date of delivery. In our study group, the main catch-up growth in weight took place after discharge until 0.5 years of corrected age.

An accelerated gain of weight within three months after term age caused an unfavourable body composition in young adulthood [3]. However, as stated above, the cohort reported by Kerkhof et al. differed from this cohort by a significantly higher gestational age and higher frequency of infants born SGA. Henriksen et al reported that severely ill ELBW infants exhibited a lower weight gain in the first months as compared to very low birth weight infants (VLBW) infants [23]. As our cohort consisted of very premature, frequently critically ill infants, we used a larger window for catch-up growth of six months.

With regards to mechanisms underlying metabolic changes, our data suggest that weight catch-up and the hereby associated metabolic risk might solely reflect the amount of protein intake during these critical first weeks of life. The risk of lifetime metabolic disturbances in ELBW children might be even more aggravated by the implementation of current guidelines, which recommend an even higher neonatal protein intake [16]. In addition, our analysis of body composition was performed in early childhood. Thus, we speculate that metabolic disturbances might even increase in the later life of these subjects

Height development differed from weight development in our cohort: After a constant decrease from birth up to 0.5 years of age, the main catch-up in height took place during the second half of the first year of life. At 9.5 years of age, target height was reached by only one-third of our cohort. Not unexpectedly, we found that the larger the change in weight-SDS from 0.5 years onwards, the higher were both fat and lean body mass as measured by DEXA in our patients. Height development only correlated with lean body mass.

The interaction between protein intake after birth and the infants’ auxological development as well as body composition later on turned out to be the focal point of our work. Protein-supplementation after birth has been discussed, in particular for extremely low birth weight infants. In our institution, protein intake for extremely low birth weight infants was usually increased from 0.5 to 1 g/kg/day on the first day of life up to a maximum of 2.5 g/kg/day (parenteral intake) and 4 g/kg/day (oral intake) in accordance to current recommendations [16,24].

It has been controversially debated whether higher protein intake during initial hospitalization directly causes uremia, acidosis and hyperammonemia [25]. Concerning long-term adverse health outcomes, protein intake may also entail a higher occurrence of long-term adverse health outcomes caused, for example, by bronchopulmonary disease (BPD) or necrotizing enterocolitis (NEC) [26]. In our cohort, we could neither verify short-term metabolic disturbances nor any occurrence of BPD or NEC which were directly affected by the amount of protein intake [8].

However, the advantages of an earlier and higher amino acid intake in form of a high protein intake, on auxological parameters were also shown by many international studies: Concerning short-term consequences, Poindexter and coworkers could demonstrate that a parenteral supplementation of amino acids of >3 g/kg/day during the first five days of life was associated with a better growth outcome at 36 postmenstrual weeks in extremely low birth weight infants compared to infants who received less amino acids [27]. At 18 months of age, however, in their study, the differences in growth parameters between groups had disappeared. A review by Premji et al. focused on studies which compared a low (<3 g/kg/day) versus a high (>3 g/kg/day but <4 g/kg/day) protein intake in low birth weight children postnatally [28]. On average, in these studies, higher protein intake resulted in an improved weight gain during the first months of life whereas gain in length was not influenced. Sufficient data about long-term auxological or cognitive outcome were missing in this review. In our cohort, average daily protein intake until discharge correlated highly significantly with main catch-up growth in weight between discharge from hospital and 0.5 years of age. However, it must be pointed out that we were not informed about the nutrition that our cohort received after discharge. In contrast to weight development, main catch-up growth in height between 0.5 and 1 years of age was not influenced by nutritional intake until discharge.

Considering long-term outcome parameters, most studies evaluated the effect of early protein administration on cognitive performance [17,18,19], whereas data on body composition of preterm infants as a result of early nutritional intake are sparse. For this purpose, dual-energy x-ray-absorptiometry is currently the most reliable method, since BMI does not accurately reflect the degree of central adiposity [29]. This is particularly important when considering that the mean BMI of our cohort at 9.5 years still remained below average. Garnett et al. revealed that mainly children with a lower birth weight and a subsequently high gain of weight afterwards tended to have a higher amount of abdominal fat in a prepubertal state [30]. Our patients were born with extremely low birth weight and frequently experienced a rapid catch-up in weight during the first six months of life. We thus hypothesize that a further increase of BMI during puberty might be associated with an even more pronounced, potentially unfavorable, fat distribution with its associated metabolic risks.

## 5. Limitations

Due to the high morbidity of our ELBW cohort and the fact that this was a single center study, our sample size was small. Furthermore, since our analysis was focused on quantity and not quality of protein intake, we were unable to analyze whether the amino acid composition of early infant nutrition might play an additional role on the long-term metabolic health of ELBW infants. On the other hand, the single center setting was advantageous with regards to the homogeneous nutritional regime and the early intensive care of our study patients. In addition, it has to be taken into account that besides early postnatal nutrition, several other pre- and postnatal factors contribute to the outcome of preterm infants but were not part of our analysis.

## 6. Conclusions

Postnatal weight and height development in extremely low birth weight infants occur during different time periods. A high amount of protein intake seems to ensure a rapid catch-up growth during the first months of life. At 9.5 years of age, however, the higher early protein supply was associated with a potentially negative impact on both body composition profile and metabolic phenotype. The higher the postnatal daily protein supply, the higher the percentage of abdominal fat mass and fasting insulin, and the lower the level of HDL-cholesterol. If confirmed in further studies, this should lead to a critical reevaluation of the nutrient regimen in low birth weight infants.

## Figures and Tables

**Table 1:** Weight, height, and body mass index (BMI) development of ELBW subjects during follow-up.

	n	Mean Weight SDS	Mean Height SDS	Mean BMI SDS
		(Range)	(Range)	(Range)
**at birth (mean GA 27.2 wks)**	61	−0.88	−0.80	
		(−2.60 to 0.44)	(−2.49 to 0.25)	
**at discharge (mean GA 40.3 wks)**	52	−2.16	−3.00	
		(−3.88 to 0.05)	(−5.19 to 1.22)	
**0.5 yrs**	61	−0.95 *	−6.56	−1.49
		(−3.15 to 1.25)	(−13.9 to 2.45)	(−5.31 to 1.64)
**1 yr**	61	−1.04	−2.28 *	−1.85
		(−3.77 to 1.31)	(−5.9 to 0.03)	(−4.83 to 1.01)
**2 yrs**	61	−1.03	−0.94 *	−1.76
		(−4.03 to 0.78)	(−3.97 to 1.75)	(−5.81 to 1.76)
**4 yrs**	61	−1.29	−1.13	−1.71
		(−3.41 to 1.23)	(−3.06 to 0.21)	(−6.5 to 1.54)
**5.7 yrs (4.5–7.7)**	61	−1.29	−0.97	−1.36
		(−4.45 to 1.04)	(−3.71 to 0.9)	(−4.55 to 1.29)
**9.5 yrs (7.9–11.9)**	39	−0.75	−0.23	−0.85
		(−3.56 to 1.76)	(−2.26 to 1.57)	(−3.71 to 1.88)

A asterisk (*) represents a change of >1 SDS between the actual and the preceding time point. GA = gestational age; SDS = standard deviation score; wks = weeks; yr = year.

**Table 2:** Stepwise linear regression analyses of body composition and metabolic parameters in former ELBW infants at 9.5 years of age (*n* = 30).

	Regression Coefficients (ß)							
Independent Variable	*Sex* ^b^	*Birth Weight* ^c^	*Early Macronutrient Intake*			*Weight Dev* ^d^	*Pubertal Stage* ^e^	*R*^2^*_corr._*
			*Carb.*	*Protein*	*Fat*			
Lean body mass (g)	−0.479 *p* = 0.003	0.476 *p* = 0.004	NS	NS	NS	NS	NS	0.44 *F* = 11.11 *p* < 0.001
Fat Mass (%)	NS	NS	NS	0.515 *p* = 0.006	NS	NS	NS	0.24 *F* = 9.05 *p* < 0.01
HDL-cholesterol (mg/dL)	NS	NS	−0.336 *p* = 0.038	−0.531 *p* = 0.002	NS	NS	NS	0.41 *F* = 10.15 *p* = 0.001
Fasting Insulin ^f^ (mU/L)	NS	NS	NS	NS	NS	0.559 *p* = 0.008	NS	0.28 *F* = 8.64 *p* < 0.01

Carb = carbohydrates; dev = development; DEXA = dual energy X-ray absorptiometry; NS = not significant. ^b^ Males had a significantly higher lean body mass than females (*p* < 0.05). ^c^ Birth weight was expressed as an SDS value. ^d^ weight development was calculated as the difference between birth weight SDS and weight SDS at the age of six months. ^e^ Pubertal stage was expressed in Tanner stages. ^f^ Fasting insulin concentrations were log-transformed before analysis.

**Table 3:** Relationship between early macronutrient intake and parameters of body composition in former ELBW subjects at 9.5 years. of age as indicated by Spearman’s correlation coefficients (*n* = 30).

	Total Fat Mass (%)	Abdominal Fat Mass (%)	Hip Fat Mass (%)	Lean Body Mass (g)	Triceps Skinfold (SDS)
**Protein intake (g/kg/day)**	***r* = 0.481**	***r* = 0.488**	***r* = 0.443**	*r* = 0.299	***r* = 0.381**
	***p* = 0.007**	***p* = 0.006**	***p* = 0.014**	*p* = 0.108	***p* = 0.05**
**Lipid intake (g/kg/day)**	*r* = 0.275	*r* = 0.157	*r* = 0.247	*r* = −0.006	*r* = 0.230
	*p* = 0.141	*p* = 0.406	*p* = 0.189	*p* = 0.975	*p* = 0.248
**Carb. Intake (g/kg/day)**	*r* = −0.036	*r* = 0.027	*r* = −0.093	*r* = −0.138	*r* = 0.117
	*p* = 0.849	*p* = 0.888	*p* = 0.624	*p* = 0.468	*p* = 0.562
**Energy intake (kcal/kg/day)**	***r* = 0.371**	*r* = 0.332	*r* = 0.319	*r* = −0.004	*r* = 0.342
	***p* = 0.043**	*p* = 0.073	*p* = 0.086	*p* = 0.984	*p* = 0.08

Carb = carbohydrate; g = grams; SDS = standard deviation score. Average carbohydrate and average lipid intake were not associated to any parameter of body composition. In addition, stepwise linear regression demonstrated that protein intake predicted fat mass (*p* < 0.05), whereas macronutrient intake did not contribute to the observed variation of lean body mass (Table 2).

**Table 4:** Association between early macronutrient and energy intake and metabolic markers in former ELBW subjects at 9.5 years of age as indicated by Spearman’s correlation coefficients (*n* = 31).

	Protein Intake	Carbohydrate Intake	Lipid Intake	Energy Intake
	(g/kg/day)	(g/kg/day)	(g/kg/day)	(kcal/kg/day)
**HDL-cholesterol (mg/dL)**	**−0.445**	**−0.417**	−0.188	**−0.393**
	***p* = 0.014**	***p* = 0.022**	*p* = 0.320	***p* = 0.032**
**LDL-cholesterol (mg/dL)**	0.143	−0.029	−0.081	−0.077
	*p* = 0.450	*p* = 0.879	*p* = 0.670	*p* = 0.722
**Total cholesterol (mg/dL)**	−0.056	−0.336	−0.169	−0.327
	*p* = 0.768	*p* = 0.070	*p* = 0.371	*p* = 0.078
**Fasting insulin (mU/L)**	**0.581**	0.155	0.077	0.232
	***p* = 0.003**	*p* = 0.469	*p* = 0.722	*p* = 0.274
**HOMA index**	**0.566**	0.152	0.073	0.230
	***p* = 0.003**	*p* = 0.468	*p* = 0.728	*p* = 0.269

## Supplementary Materials

The following are available online at http://www.mdpi.com/2072-6643/9/2/124/s1, Table S1: Partial correlation coefficients between early macronutrient intake and parameters of body composition in former ELBW subjects at 9.5 years of age, including sex as control variable, Table S2: Partial correlation coefficients between early macronutrient and energy intake and metabolic markers in former ELBW subjects at 9.5 years of age, including sex as control variable.
