# Supplementary material for: Impact of Early Nutrition on Body Composition in Children Aged 9.5 Years Born with Extremely Low Birth Weight
Source: Nutrients. 2017 Feb 10;9(2):124. doi: 10.3390/nu9020124 (PMC5331555; doi:10.3390/nu9020124)
Supplement: Supplementary file 1 [file nutrients-09-00124-s001.docx]

Supplementary Material: Impact of Early Nutrition on Body Composition in Children Aged 9.5 Years Born with Extremely Low Birth Weight

Sonja Stutte, Bettina Gohlke, Annika Peiler, Felix Schreiner, Mark Born, Peter Bartmann and Joachim Woelfle

**Table S1.** Partial correlation coefficients between early macronutrient intake and parameters of body composition in former ELBW subjects at 9.5 years of age, including sex as control variable.

|  | **Total Fat Mass (%)** | **Abdominal Fat Mass (%)** | **Hip Fat Mass (%)** | **Lean Body Mass (g)** | **Triceps Skinfold (SDS)** |
| --- | --- | --- | --- | --- | --- |
| **Protein intake** (g/kg/day) | ***r* = 0.507** | ***r* = 0.492** | ***r* = 0.512** | *r* = 0.329 | *r* = 0.098 |
|  | ***p* = 0.010** | ***p* = 0.012** | ***p* = 0.009** | *p* = 0.108 | *p* = 0.643 |
| **Lipid intake** (g/kg/day) | *r* = 0.367 | *r* = 0.319 | *r* = 0.393 | *r* = −0.251 | *r* = 0.241 |
|  | *p* = 0.071 | *p* = 0.120 | *p* = 0.052 | *p* = 0.226 | *p* = 0.246 |
| **Carb. Intake** (g/kg/day) | *r* = −0.099 | *r* = 0.143 | *r* = −0.043 | *r* = −0.235 | *r* = 0.172 |
|  | *p* = 0.639 | *p* = 0.495 | *p* = 0.840 | *p* = 0.258 | *p* = 0.412 |
| **Energy intake** (kcal/kg/day) | *r* = 0.359 | *r* = 0.355 | *r* = 0.341 | *r* = −0.056 | *r* = 0.265 |
|  | *p* = 0.078 | *p* = 0.081 | *p* = 0.095 | *p* = 0.789 | *p* = 0.20 |

Carb = carbohydrate; g = grams; SDS = standard deviation score.

**Table S2.** Partial correlation coefficients between early macronutrient and energy intake and metabolic markers in former ELBW subjects at 9.5 years of age, including sex as control variable.

|  | **Protein Intake (g/kg/Day)** | **Carbohydrate Intake (g/kg/Day)** | **Lipid Intake (g/kg/Day)** | **Energy Intake (kcal/kg/Day)** |
| --- | --- | --- | --- | --- |
| **HDL cholesterol (mg/dL)** | **−0.365** | **−0.578** | **−**0.248 | **−0.579** |
|  | ***p* = 0.043** | ***p* = 0.002** | *p* = 0.127 | ***p* = 0.002** |
| **LDL cholesterol (mg/dL)** | −0.101 | −0.165 | −0.154 | −0.208 |
|  | *p* = 0·323 | *p* = 0·226 | *p*= 0·242 | *p* = 0·170 |
| **Total cholesterol (mg/dL)** | −0.249 | **−0.482** | −0.335 | **−0.535** |
|  | *p* = 0.126 | ***p* = 0.010** | *p* = 0.059 | ***p* = 0.004** |
| **Fasting insulin (mU/L)** | **0.423** | 0.126 | 0.235 | 0.279 |
|  | ***p* = 0.022** | *p* = 0.284 | *p* = 0.140 | *p* = 0.099 |
| **HOMA index** | **0.482** | 0.145 | 0.224 | 0.297 |
|  | ***p* = 0.010** | *p* = 0.254 | *p* = 0.153 | *p* = 0.085 |
